# Maternal depressive symptoms trajectories and harsh parenting: the mediating role of maternal quality of life in the 2004 Pelotas Birth Cohort

**DOI:** 10.47626/1516-4446-2024-3652

**Published:** 2025-01-22

**Authors:** Alicia Matijasevich, Jessica Mayumi Maruyama, Luciana Tovo-Rodrigues, Iná S. Santos

**Affiliations:** 1 Departamento de Medicina Preventiva, Faculdade de Medicina, Universidade de São Paulo, São Paulo, SP, Brazil; 2 Programa de Pós-Graduação em Epidemiologia, Universidade Federal de Pelotas, Pelotas, RS, Brazil; 3 Programa de Pós-Graduação em Ciências do Desenvolvimento Humano, Universidade Presbiteriana Mackenzie, São Paulo, SP, Brazil

**Keywords:** Maternal depression, child maltreatment, quality of life, mediation, cohort

## Abstract

**Objective:**

To study the impacts of maternal depressive symptoms trajectories and harsh parenting and explore if the maternal quality of life (QoL) mediates this association.

**Methods:**

We used data from the 2004 Pelotas Birth Cohort, a population-based longitudinal study from Pelotas, Brazil (n=3,285 mothers, complete cases analysis). We used the Edinburgh Postnatal Depression Scale (EPDS) to assess maternal depressive symptoms and calculated their trajectories from 3 months until the 11-year follow-up using a group-based modeling approach. Psychological and physical aggression were measured using the Parent-Child Conflict Tactics Scale (CTSPC). Maternal QoL was measured by the question “How is your quality of life?” Data were analyzed using path models in Mplus.

**Results:**

All maternal depressive symptoms trajectories increased the frequency of psychological and physical aggression at early adolescence when compared to the reference group. Mediation analysis indicated that maternal depressive symptoms led to low levels of perceived maternal QoL, which in turn was associated with increased use of harsh parenting. The proportion of total effect explained by maternal QoL ranged from 4.04% (0.00-5.58%) to 16.31% (10.88-19.10%).

**Conclusion:**

Our findings, within a longitudinal framework from a middle-income country, support existing evidence that maternal depressive symptoms are associated with harsh parenting. Our results also suggest that one mechanism underlying this association is lower perceived maternal QoL.

## Introduction

Harsh parenting practices are prevalent worldwide and represent serious public health concerns.^1^ Reports on global lifetime prevalence estimate that nearly 36% of children were psychologically abused, 23% were physically abused, 13% were sexually abused, 16% suffered physical neglect, and 18% emotional neglect.^1^,^2^ Numerous studies have shown that experiencing any kind of maltreatment by parents or caregivers severely impacts children’s development, mental and physical health with long-term consequences.^3^-^5^ Child abuse is associated with altered brain architecture, cognitive and academic under-performance, socioemotional impairments, substance abuse, engagement in risky behaviors, involvement in delinquency, and increased risk of developing psychiatric disorders.^3^-^5^ The adverse impacts of childhood maltreatment also have costly implications for society due to their effects on workforce and human capital development, as well as their burden on the healthcare and criminal justice systems.^5^,^6^

The literature on harsh parenting has identified a multitude of risk factors whereby its occurrence involves a complex, dynamic interplay between those factors at the individual, family, and societal levels.^1^,^7^ Characteristics of the child that have been associated with an increased likelihood of victimization include being either under 4 years old or adolescent, having any physical or mental disability, being a child of an unplanned pregnancy, and presenting a behavioral problem, such as externalizing symptoms.^1^,^8^ Previous research also indicates gender differences in child maltreatment, where girls may be more vulnerable to sexual abuse whereas boys may be more likely to experience physical aggression.^1^ Family factors and parent-related characteristics associated with child maltreatment include parental low self-esteem, mental health and substance use problems, history of childhood abuse, high levels of family conflict and parents’ stress, poor family cohesion, and the presence of intimate partner violence.^7^,^9^ Socioeconomic disadvantages, such as low parental education, low family income, and teenage parenthood, also elevate risk of engagement in maltreating behavior.^7^,^10^,^11^ Societal and cultural aspects that have been linked to childhood abuse or neglect include neighborhood deprivation, poor social capital, lack of access to child daycare centers, and cultural acceptance of physical punishment as a method of disciplining children.^1^,^7^,^11^ Harsh parenting is usually not an isolated event; a maltreated child faces a six-fold increased risk of suffering recurrent episodes compared to a child who has never been maltreated.^9^ Parent-child relationship difficulties, parental substance use, and mental health problems were found to be consistent predictors of maltreatment recurrence.^9^

Maternal depression is a common psychiatric disorder, affecting approximately 20% of women worldwide, and has a huge burden on women’s and child’s health and wellbeing.^12^ As mentioned, evidence has shown that maternal depression is associated with a greater chance of perpetrating physical aggression, psychological abuse, or any form of neglect.^1^,^13^-^15^ In addition, after a first episode of depressive symptoms, a woman is at an increased risk of presenting subsequent and chronic depressive episodes, which, in turn, can lead to multiple and recurrent child-abusing or neglecting behaviors.^13^-^15^ While evidence suggests a recurrent pattern of maternal depressive symptoms, few studies have utilized a longitudinal design to explore its association with child maltreatment or harsh parenting, particularly in low- and middle-income countries (LMICs).^16^ Moreover, most previous research has measured child maltreatment or harsh parenting in early childhood; the early adolescence period is poorly investigated.^14^

There are several potential mechanisms linking maternal depressive symptoms and harsh parenting behaviors. Depressive mothers are more likely to engage in maltreating behaviors as they frequently experience more parenting difficulties, such as less emotional sensitivity, more hostile interactions, irritability toward children, and inconsistent responsiveness to the child’s needs.^17^,^18^ Many of the most prevalent symptoms of depression, such as fatigue, lack of interest in daily activities, sleep problems, and feelings of worthlessness, can directly impact perceived quality of life (QoL), reducing these women’s ability to manage the care of their infants.^17^,^18^ In fact, some evidence has shown that depressed mothers report lower overall QoL than non-depressed mothers.^19^ QoL is a multifaceted concept that encompasses an individual’s overall well-being, including physical health, psychological state, level of independence, social relationships, personal beliefs, and interaction with key aspects of their surroundings, such as the home, neighborhood, and access to resources.^20^ Maternal QoL specifically pertains to a mother’s well-being in these domains as influenced by the demands and experiences of motherhood. The association between maternal QoL and maternal depression is significant, as depression can severely impair a mother’s physical and psychological health, social interactions, and ability to engage in daily activities, thereby diminishing her overall QoL.^20^,^21^ Conversely, poor QoL can exacerbate depressive symptoms, creating a cyclical relationship where each factor negatively impacts the other.^20^,^21^ Moreover, maternal depression and low QoL are often linked to harsh parenting practices, as the stress and emotional challenges faced by mothers can affect their parenting behaviors.^21^-^23^ Conducting a study to examine the relationship between maternal depressive symptoms, maternal QoL, and harsh parenting is relevant for both theoretical and practical reasons. Theoretically, understanding this complex interplay can enrich psychological and developmental theories by providing evidence on the mechanisms linking mental health, well-being, and parenting practices. Practically, the findings can inform interventions aimed at improving maternal QoL (e.g., social support interventions), which in turn could mitigate depressive symptoms and reduce harsh parenting behaviors, ultimately fostering better outcomes for both mothers and children.

While several studies have examined the relationship between maternal depression and parenting behaviors, to the best of our knowledge, few have focused on the mediating role of QoL.^19^,^21^ Additionally, there is a lack of research in LMICs like Brazil, where socioeconomic factors and healthcare access differ significantly from those of high-income countries.^24^ Our study addresses this gap by providing insights into these relationships within the Brazilian context, contributing to the global understanding of maternal mental health and parenting practices. Using data from a large, ongoing Brazilian population-based cohort, we aimed to study the association of maternal depressive symptoms trajectories and harsh parenting (psychological and physical aggression) at age 11, controlling for potential confounder variables. Additionally, we aimed to explore whether maternal perceived QoL would mediate the association of interest. We hypothesize that a high-chronic trajectory of depressive symptoms will be associated with more frequent use of psychological and physical aggression and that a portion of these effects will be mediated by the woman’s perceived QoL.

## Methods

### Participants

This study used data from the 2004 Pelotas Birth Cohort, a population-based longitudinal study. Pelotas is a medium-sized city in the state of Rio Grande do Sul, located in the south of Brazil, with an estimated population of nearly 340,000 as of 2004 (93.2% were urban residents). The cohort recruited 4,231 live newborns, corresponding to 99.2% of all births occurring in the city in 2004. Mothers were interviewed within 24 hours postpartum using a standardized questionnaire. Mothers and their children were assessed again at home at mean (SD) ages of 3.0 (0.1), 11.9 (0.2), 23.9 (0.4), and 49.5 (1.7) months and at a research clinic when participants were 6.8 (0.3) and 11.0 (0.3) years old. The follow-up rates ranged from 86.6 to 99.2%. Details of the cohort and data collection can be found elsewhere.^25^,^26^

### Measures

#### Maternal depressive symptoms trajectories

The Edinburgh Postnatal Depression Scale (EPDS) is a self-report 10-item scale, each item having four possible responses from 0 to 3, which assess the intensity of depressive symptoms over the previous 7 days.^27^ The score ranges from 0 to 30 points, with higher scores indicating more severe depressive symptoms. We used a previously translated and validated version of the EPDS.^28^

The trajectories of maternal depressive symptoms were constructed using the EPDS scores from the 3-month to the 11-year-old follow-up through a semiparametric group-based modelling approach.^29^,^30^ The number and shape of trajectories were based on the best fit of the model (maximum Bayesian information criteria [BIC]), interpretability of the trajectories obtained, and on the posterior probability scores for each trajectory group.^29^,^30^ Details of the steps and methods used to identify the trajectories of maternal depressive symptoms can be found elsewhere.^30^,^31^

A five-group trajectories model emerged as best-fitting and most parsimonious: Groups 1 (low) and 2 (moderate-low) (74.5% of the sample) included women with EPDS < 10 across all time points. Group 3, “increasing” (11.1%), represented those women who had low levels of depressive symptoms in their children’s first years of life followed by a consistent increase in EPDS scores during the study period. The fourth group, “decreasing” (9.3%), was composed of women that showed high EPDS scores in the first 2 years postpartum and decreasing symptoms afterwards. The fifth group, the “high-chronic” trajectory, comprised 5.2% of the sample and included mothers with high EPDS scores (> 15 points) during all 11 years of their children’s life. For all five groups, the average posterior probability was above the lower recommended threshold for assignment of 0.7 (average posterior probability of 0.87, 0.81, 0.78, 0.79, and 0.87 for Groups through 5, respectively).^29^,^30^

#### Harsh parenting at age 11 years

We used the parent-report version of the Parent-Child Conflict Tactics Scale^32^ (CTSPC) to assess harsh parenting at age 11 years. The CTSPC questionnaire is widely used in epidemiological and clinical studies to capture the frequency of negative parental conflict behaviors of child physical and emotional maltreatment during the preceding year.^28^ In the current study, we used a 14-item version consisting of two subscales: i) psychological aggression, related to emotional maltreatment, for example, “How many times did you shout, yell, or screamed at him/her” (five items), and ii) physical aggression (nine items), encompassing items related to physical punishment (six items) and physical maltreatment (three items) (e.g., “How many times did you hit him/her with a fist or kick him/her hard?”). Each item was rated on a Likert scale as 0 (never in the past year), 1 (once in the past year), or 2 (more than once in the past year).

#### Maternal perceived quality of life

Maternal perceived QoL was measured by the question “How is your quality of life?” with five possible answer options (very poor, poor, fair, good, very good) in the 11-year follow-up. Higher scores indicate worse perceived QoL.

#### Covariates

The covariates were collected in the perinatal interview and include monthly household income in the month before delivery (quintiles), maternal education (number of completed years of formal education), maternal age at childbirth, maternal self-reported skin color (white or non-white), marital status (mothers were asked if they were living with a partner; yes/no), and the child’s sex.

### Statistical analysis

The mean and SD scores of psychological and physical aggression subscales according to family and child characteristics were analyzed using analysis of variance (ANOVA). We used an indicator coding system to represent the different groups for the main exposure (maternal depressive symptoms trajectories).^33^ Briefly, dummy variables were created for each category of exposure (low, moderate-low, increasing, decreasing, and high-chronic trajectories), in which cases were set to 1 in that category, and 0 otherwise. The group that was not explicitly coded was treated as the reference category in the analysis (in our study, the low depressive symptoms trajectory), and the parameters in the model related to group differences were quantifications relative to this reference group.

**Figure 1:**
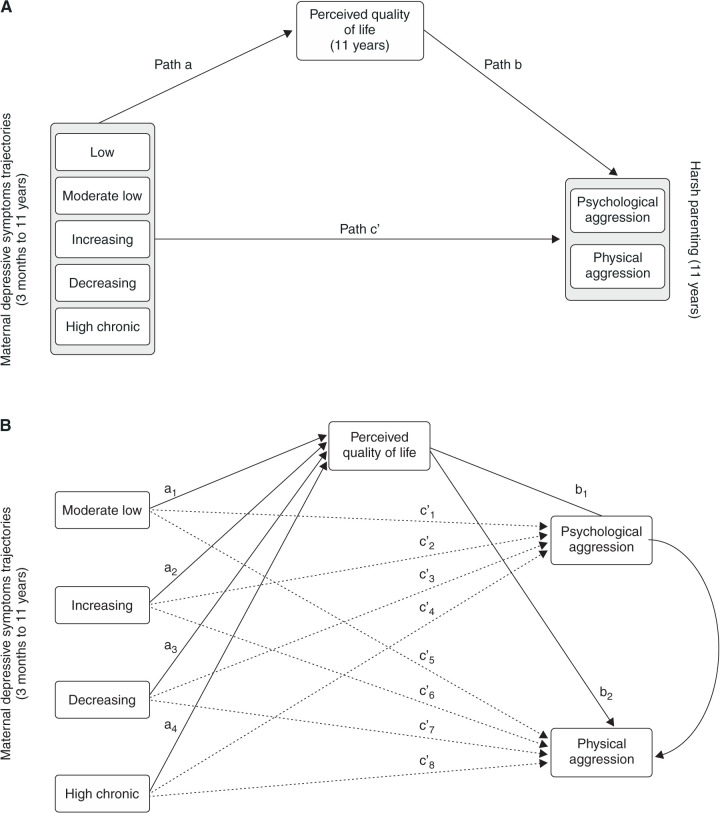
Conceptual and statistical model for the association between maternal depressive symptoms and harsh parenting mediated by perceived quality of life (QoL). A) Conceptual model. The harsh parenting subscales were included in the same model. B) Final statistical model. Dotted lines represent the direct pathways (i.e., the effects of maternal depression on CTSPC scales not mediated by QoL). Solid lines represent the indirect pathways (i.e., the effects of maternal depression on CTSPC scales mediated by QoL). The two-headed arrow represents the covariance between the psychological and physical aggression scales (r = 0.756, standard error [SE] = 0.034, p < 0.001).

We used a path model to examine the mediating role of perceived QoL in the relationship between maternal depressive symptoms trajectories and the CTSPC subscales (psychological aggression and physical aggression). Figure 1A represents the conceptual model, showing the effects of each trajectory of maternal depressive symptoms relative to the reference group on child-disciplining tactics. The total effect (path c) comprises the direct effect pathway (path c’) of each maternal depression trajectory on CTSPC subscales and the indirect effect pathway through the perceived QoL (path a*b). The mediating effect was considered significant if the 95% bias-corrected CI based on 5,000 bootstrap samples did not include zero.^33^,^34^ All pathways were controlled for the covariates (family income, maternal education, maternal skin color, marital status, maternal age, and child’s sex). The proportion explained was calculated when both the indirect and total effects were statistically significant, indicating the proportion of the total effect attributable to the mediator.^35^ Descriptive statistics were conducted using STATA version 14.2 and path analyses were conducted using Mplus version 8.0 software. Due to moderate skewness on all CTSPC scales, all models were estimated using maximum likelihood estimation (MLE) and bootstrapped standard errors, which produces standard errors which are robust to non-normality.^36^,^37^ All statistical tests were two-tailed, and significance was determined at the 0.05 level.

### Ethics statement

All 2004 Pelotas Birth Cohort follow-ups were approved by the research ethics committee of Faculdade de Medicina, Universidade Federal de Pelotas. All mothers or principal caregivers of the participating children signed an informed consent form before data collection. At age 11 years, adolescents signed an informed consent form as well.

## Results

### Attrition analysis

For the current study, we used the complete cases analysis approach. Therefore, we excluded those individuals with missing values for the exposure (maternal depressive symptoms trajectories), the mediator (maternal perceived QoL), and the outcome (CTSPC scales). In addition, we included only maternal reports of CTSPC scales (biological and adoptive mothers, n=3,322, 94.6%). The final sample size comprised n=3,285 observations. Table 1 shows the comparison between those included and not included in the analysis regarding socioeconomic variables. Women included in the current study has a higher household income, completed more years of education, were older, and were more often living with a partner at childbirth.

### Sample characteristics

CTSPC scale scores according to socioeconomic and family characteristics are presented in Table 2. More frequent use of psychological and physical aggression was reported in families from disadvantaged socioeconomic status, i.e., those with lower income, low maternal schooling, single, young, and non-white mothers. Physical aggression was also more frequent with boys.

Mothers belonging to the high-chronic depressive symptoms trajectory reported more frequent use of psychological and physical aggression when compared to mothers in the low depressive symptoms trajectory. In addition, mothers with very poor QoL also reported more frequent use of harsh parenting (Table 2).

### Associations between maternal depressive symptoms trajectories, perceived quality of life, and psychological and physical aggression

Table 3 shows the associations between the maternal depressive symptoms trajectories and the perceived QoL (paths a1 to a4 in Figure 1B) and between the perceived QoL and psychological (path b1) and physical aggression (path b2). Relatively to the low trajectory group, all trajectories of maternal depressive symptoms predicted a worse perceived QoL. In turn, higher scores on the QoL scale were associated with less frequent use of psychological and physical aggression (Table 3).

### Total effects of maternal depressive symptoms trajectories on the frequency harsh parenting

All elevated maternal depressive symptoms trajectories were associated with more frequent use of psychological and physical aggression (Tables 4 and 5 and Figure 1B). Women belonging to the high-chronic depressive symptoms trajectory reported a mean score of 0.997 (95%CI 0.778-1.164) points higher in the psychological aggression scale when compared to women in the low depressive symptoms trajectory. For physical aggression scales, the mean scores were 0.950 (95%CI 0.570-1.272) points higher for women in the high-chronic trajectory when compared to the reference category.

### Mediating effects of perceived quality of life

Tables 4 and 5 and Figure 1B show the direct and indirect effects of maternal depressive symptoms trajectories on CTSPC scales via maternal perceived QoL. All the relative indirect effects of maternal depression trajectories on the use of psychological and physical aggression through the perceived QoL were positive and significant, indicated by the 95% bias-corrected bootstrap CI that did not include zero, supporting the hypothesis of a mediated-effect through the perceived QoL (Tables 4 and 5). These findings show that maternal depressive symptoms were associated with more frequent use of psychological and physical aggression towards the child and that an underlying mechanism explaining these effects could be a worse QoL experienced by the women. The proportion of total effect explained by maternal QoL ranged from 4.04% (0.00-5.58%; moderate-low depressive symptoms trajectory and psychological aggression) to 16.31% (10.88-19.10%; high-chronic depressive symptoms trajectory and physical aggression).

## Discussion

This study assessed the impacts of maternal depressive symptoms trajectories on the frequency of harsh parenting in a prospective population-based birth cohort from Brazil. In addition, we explored the mediating role of maternal perceived QoL in the aforementioned association. Our findings indicate that elevated maternal depressive symptoms trajectories from the child ages of 3 months through 11 years increased the frequency of psychological and physical aggression at early adolescence. Moreover, we found that part of these effects was mediated by impaired maternal QoL.

The use of harsh parenting behavior was associated with socioeconomic disadvantages, such as low income, low maternal schooling, and single parenthood. A recent umbrella synthesis of meta-analyses on child maltreatment and harsh presenting antecedents and preventive interventions reported that a low socioeconomic status is a critical predictor of elevated risk of harsh parenting, and policies aiming to reduce the economic difficulties in low-income families (e.g., cash transfer) may represent an effective way of reducing and preventing child maltreatment.^38^ The transmission of child physical and psychological aggressions across generations was one the most robust effects that emerged from the synthesis, highlighting the relevance of parental experience of childhood abuse in perpetrating maltreatment in offspring.^38^ Unfortunately, data on maternal history of childhood maltreatment were not available in our dataset, and thus we were unable to examine the intergenerational cycle of abuse in the present study. However, our findings contribute to the existing evidence by investigating the effects of maternal depressive symptoms on harsh parenting and a mediation model that explains this association. Both aspects were identified as gaps in the current meta-analytic literature by van IJzendoorn et al.^38^

These results are in line with existing studies showing that maternal depressive symptoms are associated with child maltreatment and harsh parenting occurrence.^1^,^13^-^15^ Our findings extend the literature by showing that even mothers with mild depressive symptoms (i.e., belonging to the moderate-low depressive symptoms trajectory) exhibited increased use of physical and psychological aggression as a disciplinary strategy when compared to mothers in the reference group. A further interesting finding was that even mothers in the decreasing trajectories (i.e., those whose symptoms improved over time) exhibited more harsh parenting behaviors, suggesting that early depressive symptoms may have a lasting impact on parenting practices. Kuckertz et al.^39^ showed that depressed mothers were more likely to be psychologically aggressive toward their children, which was in turn associated with higher child internalizing symptoms. Wolford et al.^40^ also reported that maternal depressive symptoms increased the use of harsh parenting practices, which, in turn, were associated with greater internalizing and externalizing symptoms in the youth. This greater risk of harsh parenting perpetration by depressed mothers may explain part of the intergenerational transmission of psychopathologies.^41^ In addition, Choi et al.^15^ reported the effects of postpartum depression on child maltreatment and found that subsequent child mental health problems were influenced by later maternal depression. This result highlights the importance of considering the persistent nature of maternal depression beyond the postpartum period, as examined by our study. Furthermore, while not the focus of this investigation, it must be acknowledged that child maltreatment also presents a recurrent pattern, i.e., maltreated children are at greater risk of experiencing subsequent maltreatment.^9^ Future work should consider including repeated measures of child maltreatment or harsh parenting to explore cumulative and interactive effects of harsh parenting and maternal depression on adverse child outcomes.

We also found that all elevated maternal depressive symptoms trajectories were associated with worse perceived maternal QoL. Indeed, a recent systematic review showed that depressed pregnant and postpartum women presented lower levels of QoL when compared to their nondepressed counterparts.^42^ It has been reported that, although the severity of depressive symptoms and worst QoL are highly correlated, changes in QoL are not fully explained by changes in depression levels.^43^ Moreover, treatments can effectively reduce clinical-rated depressive symptoms but may not promote improvement in a patient’s perception of QoL.^43^ Taken together, decreases in QoL are not necessarily present in depressed mothers, and the improvement of QoL can also be related to optimized long-term management of maternal depressive symptoms.^15^ In addition, our findings also suggest that part of the negative effects of maternal depression on the frequency of harsh parenting were mediated by lower levels of QoL, showing that QoL is relevant not only for women’s well-being but also for their parenting behavior. A meta-analysis of effective components of child maltreatment interventions reported that addressing parental mental health problems emerged as an important factor in reducing harsh parenting practices.^44^ Parental QoL improvement may also represent a possible way of reducing parental abusive behavior aiming to prevent child maltreatment and harsh parenting, which justifies further investigation.

The strengths of our study include the longitudinal design, large sample size, and high retention rate from an upper-middle-income country (Brazil). Unlike most previous work, maternal depression was evaluated through developmental trajectories, which allowed for the examination of different patterns of symptoms over time and identified differences in harsh parenting between trajectory groups. In addition, we contribute to the literature by assessing harsh parenting in early adolescence. Some limitations, however, warrant consideration. The 20% loss to follow-up in our study raises the possibility of selection bias. To address potential confounding, baseline variables were included as controlling variables in our mediating analysis. Despite these adjustments, unmeasured factors could still affect our results. Therefore, our findings should be interpreted with caution, acknowledging that this attrition might underrepresent certain subgroups and potentially lead to an underestimation of the associations of interest. The mediator was based on one single, general question about women’s perception of their QoL. QoL is a broad construct that includes several domains, including physical, environmental, and social interactions, among others.^45^ The utilization of a full scale encompassing the complexity of all facets of QoL might offer more informative insights, particularly in the presence of depressive symptoms, and should be considered in future studies. Furthermore, it is important to note that the effects on the perceived QoL are likely more associated with certain types of depression symptoms, such as hopelessness. However, due to the lack of detailed data on specific symptoms of maternal depression, we were unable to evaluate whether changes in QoL would vary according to different types of maternal depression. Similarly, harsh parenting was assessed solely through maternal reports, which may be biased regarding how children perceive the aggression committed by caregivers.^46^ However, this concern can be mitigated as we did not assess children’s outcomes (e.g., children’s mental health outcomes). A further limitation of our study is that we focused exclusively on one mechanism linking maternal depressive symptoms and harsh parenting. Future studies should explore additional variables that may influence this association to provide a more comprehensive understanding. Finally, while we controlled for relevant measured confounders in all pathways, mediation analyses rely on strong assumptions related to unmeasured confounders, and their presence cannot be entirely ruled out.

The findings from the present study support existing evidence indicating that maternal depressive symptoms are associated with the use of harsh parenting practices towards early adolescents, within a longitudinal framework. Our study highlights the role of maternal QoL as a mediator in the relationship between maternal depression and harsh parenting behaviors. These findings contribute to our understanding of how improving QoL (e.g., through social support interventions) can potentially mitigate the negative impact of maternal depression on parenting practices. Future research should continue to explore other potential mediators and moderators to further elucidate the complex pathways involved.

## Disclosure

The authors report no conflicts of interest.

## Figures and Tables

**Table 1:** Socioeconomic differences between the individuals included and not included in the current study

Variables	Not included (n=946)	Included (n=3,285)	p-value
Household income (quintiles)			< 0.001
1st (poorest)	259 (27.4)	613 (18.7)	
2nd to 5th	687 (72.6)	2,672 (81.3)	
Maternal schooling (years)			< 0.001
0-4	183 (19.6)	472 (14.5)	
5-8	399 (42.7)	1,332 (40.9)	
≥ 9	353 (37.7)	1,449 (45.5)	
Maternal skin color			0.625
White	685 (72.4)	2,405 (73.2)	
Non-white	261 (27.6)	880 (26.8)	
Living with a partner			< 0.001
Yes	752 (79.5)	2,784 (84.7)	
No	194 (20.5)	501 (15.2)	
Maternal age at childbirth (years)			< 0.001
< 20	276 (29.2)	758 (23.1)	
21-34	587 (62.1)	2,166 (65.9)	
≥ 35	82 (8.7)	360 (11.0)	
Child’s sex			0.605
Male	498 (52.6)	1,698 (51.9)	
Female	448 (47.4)	2,035 (48.1)	

Data presented as n (%).

The results were derived using the chi-square test.

**Table 2:** Mean (SD) scores of psychological aggression and physical aggression according to family and child characteristics

	Psychological aggression	Physical aggression
	(range: 0-5)	(range: 0-9)
Household income (quintiles)	p < 0.001	p < 0.001
1 (lowest)	2.19 (1.13)	1.73 (1.81)
2	2.23 (1.06)	1.71 (1.81)
3	2.20 (1.09)	1.59 (1.72)
4	2.11 (1.11)	1.53 (1.72)
5 (highest)	1.92 (1.06)	1.23 (1.53)
Maternal schooling (years)	p < 0.001	p < 0.001
0-4	1.98 (1.19)	1.61 (1.77)
5-8	2.28 (1.10)	1.74 (1.84)
≥ 9	2.05 (1.04)	1.37 (1.58)
Maternal skin color	p = 0.054	p < 0.001
White	2.11 (1.09)	1.46 (1.67)
Non-white	2.19 (1.10)	1.83 (1.85)
Living with a partner	p < 0.001	p < 0.001
Yes	2.09 (1.09)	1.50 (1.69)
No	2.36 (1.08)	1.87 (1.86)
Maternal age (years)	p < 0.001	p < 0.001
< 20	2.41 (1.04)	1.94 (1.88)
20-34	2.09 (1.10)	1.48 (1.68)
≥ 35	1.79 (1.08)	1.26 (1.51)
Child’s sex	p = 0.492	p < 0.001
Male	2.14 (1.08)	1.70 (1.79)
Female	2.12 (1.12)	1.41 (1.64)
Maternal depressive trajectories	p < 0.001	p < 0.001
Low	1.75 (1.07)	1.14 (1.45)
Moderate-low	2.21 (1.03)	1.64 (1.69)
Increasing	2.47 (1.07)	2.06 (1.97)
Decreasing	2.39 (1.06)	1.80 (1.88)
High-chronic	2.77 (1.18)	2.14 (2.14)
Perceived quality of life	p < 0.001	p < 0.001
Very good	1.87 (1.10)	1.27 (1.56)
Good	2.08 (1.08)	1.47 (1.67)
Fair	2.40 (1.08)	1.95 (1.85)
Poor	2.41 (1.17)	2.38 (2.09)
Very poor	2.65 (1.13)	1.92 (2.41)

The results were derived using the analysis of variance (ANOVA) test. Higher scores on Parent-Child Conflict Tactics Scale (CTSPC) subscales indicate that psychological and physical aggression were more frequent.

**Table 3:** Associations between the main exposure (maternal depressive symptoms trajectories) and the mediator (perceived QoL) and between the mediator and the outcomes (psychological and physical aggression)

Maternal depressive symptoms trajectories → perceived QoL	B (95%CI)	p-value
Low	0.000 (reference)	< 0.001
Moderate-low (a_1_)	0.166 (0.120-0.219)	< 0.001
Increasing (a_2_)	0.575 (0.484-0.661)	< 0.001
Decreasing (a_3_)	0.277 (0.205-0.359)	< 0.001
High-chronic (a_4_)	0.914 (0.781-1.064)	< 0.001
Perceived QoL → psychological aggression (b_1_)	0.107 (0.040-0.158)	< 0.001
Perceived QoL → physical aggression (b_2_)	0.169 (0.059-0.258)	0.001

The results were derived using separate multivariate linear regressions for each outcome separately. All analyses were adjusted for the confounders (household income, maternal schooling, maternal skin color, maternal age, marital status, and child’s sex).

B = unstandardized coefficients; QoL = quality of life.

**Table 4:** Relative indirect, direct, and total effects of maternal depressive symptoms trajectories on psychological aggression mediated by perceived QoL (reference group: low trajectory; n=3,285)

Maternal depression trajectories/relative effects†	B (SE)‡	95% bias-corrected bootstrap CI	Proportion explained (%) by maternal QoL
Moderate-low			
Total effect	0.445*	0.369-0.538	4.04 (0.00-5.58)
Direct effect (c’_1_)	0.427*	0.350-0.520	
Indirect effect (a_1_*b_1_)	0.018*	0.008-0.030	
Increasing			
Total effect	0.689*	0.577-0.820	8.99 (3.99-11.22)
Direct effect (c’_2_)	0.627*	0.494-0.764	
Indirect effect (a_2_*b_1_)	0.062*	0.023-0.092	
Decreasing			
Total effect	0.601*	0.461-0.758	4.99 (2.60-6.33)
Direct effect (c’_3_)	0.572*	0.436-0.741	
Indirect effect (a_3_*b_1_)	0.030*	0.012-0.048	
High-chronic			
Total effect	0.997*	0.778-1.164	9.83 (4.75-12.89)
Direct effect (c’_4_)	0.899*	0.678-1.088	
Indirect effect (a_4_*b_1_)	0.098*	0.037-0.150	

B = unstandardized coefficient; QoL = quality of life; SE = standard error.

† Relative effect to the reference group (low depressive symptoms trajectory).

‡ Estimates for the fully adjusted model including household income, maternal education, maternal skin color, maternal age, marital status, and child’s sex in all pathways.

* p < 0.001.

**Table 5:** Relative indirect, direct, and total effects of maternal depressive symptoms trajectories on physical aggression mediated by perceived QoL (reference group: low trajectory; n=3,285)

Maternal depression trajectories/relative effects†	B (SE)‡	95% bias-corrected bootstrap CI	Proportion explained (%) by maternal QoL
Moderate-low			
Total effect	0.470*	0.353-0.593	5.95 (3.39-7.92)
Direct effect (c’_1_)	0.442*	0.326-0.567	
Indirect effect (a_1_*b_1_)	0.028**	0.012-0.047	
Increasing			
Total effect	0.838*	0.606-1.057	11.57 (6.10-14.38)
Direct effect (c’_2_)	0.741*	0.502-0.954	
Indirect effect (a_2_*b_1_)	0.097*	0.037-0.152	
Decreasing			
Total effect	0.583*	0.368-0.839	8.06 (5.16-9.65)
Direct effect (c’_3_)	0.537*	0.328-0.810	
Indirect effect (a_3_*b_1_)	0.047**	0.019-0.081	
High-chronic			
Total effect	0.950*	0.570-1.272	16.31 (10.88-19.10)
Direct effect (c’_4_)	0.796*	0.417-1.127	
Indirect effect (a_4_*b_1_)	0.155*	0.062-0.243	

B = unstandardized coefficient; QoL = quality of life; SE = standard error.

† Relative effect to the reference group (low depressive symptoms trajectory).

‡ Estimates for the fully adjusted model including household income, maternal education, maternal skin color, maternal age, marital status, and child’s sex in all pathways.

* p < 0.001; ** p < 0.05.
